# The Role of Mesh Implants in Surgical Treatment of Parastomal Hernia

**DOI:** 10.3390/ma14051062

**Published:** 2021-02-24

**Authors:** Karolina Turlakiewicz, Michał Puchalski, Izabella Krucińska, Witold Sujka

**Affiliations:** 1 Institute of Material Science of Textiles and Polymer Composites, Lodz University of Technology, Żeromskiego 116, 90-924 Lodz, Poland; michal.puchalski@p.lodz.pl (M.P.); izabella.krucinska@p.lodz.pl (I.K.); 2 Tricomed S.A., Świętojańska 5/9, 93-493 Lodz, Poland; witold.sujka@tzmo-global.com

**Keywords:** parastomal hernia, surgical mesh, hernia repair, prevention, biocompatibility

## Abstract

A parastomal hernia is a common complication following stoma surgery. Due to the large number of hernial relapses and other complications, such as infections, adhesion to the intestines, or the formation of adhesions, the treatment of hernias is still a surgical challenge. The current standard for the preventive and causal treatment of parastomal hernias is to perform a procedure with the use of a mesh implant. Researchers are currently focusing on the analysis of many relevant options, including the type of mesh (synthetic, composite, or biological), the available surgical techniques (Sugarbaker’s, “keyhole”, or “sandwich”), the surgical approach used (open or laparoscopic), and the implant position (onlay, sublay, or intraperitoneal onlay mesh). Current surface modification methods and combinations of different materials are actively explored areas for the creation of biocompatible mesh implants with different properties on the visceral and parietal peritoneal side. It has been shown that placing the implant in the sublay and intraperitoneal onlay mesh positions and the use of a specially developed implant with a 3D structure are associated with a lower frequency of recurrences. It has been shown that the prophylactic use of a mesh during stoma formation significantly reduces the incidence of parastomal hernias and is becoming a standard method in medical practice.

## 1. Introduction

Hernias are one of the most common diseases treated by surgery and occur in both women and men, regardless of age. The formation of a hernia is the result of intra-abdominal pressure that is greater than the strength of the connective tissues [1]. A typical hernia consists of a gate and a hernial sac and its contents, e.g., an intestinal loop or a fragment of the network.

The appearance of hernias is facilitated by factors that weaken the transverse fascia or increase the pressure on it, disturbances in collagen metabolism, and defects in anatomical structures [2].

Additionally, some diseases promote the development of hernias, including prostate hypertrophy, malnutrition, constipation, diabetes, hypoproteinemia, or previous surgical procedures that weakened the strength of the connective tissue [3].

Post-operative infection and recurrence are the primary problems associated with hernia repair [4]. The formation of peritoneal adhesions between the abdominal viscera and the mesh is another cause for concern and is the most crucial parameter for parastomal hernia repair. Mesh applications also remain a concern due to potential mesh-associated complications [5]. The selection of an appropriate mesh type and composition of biomaterials affects the success post-implantation and the ability to avoid complications related to specific surgeries [6]. The ideal mesh must permit repair of the fascial defect and incorporation into the surrounding body tissue while providing little adhesion, minimal immune reactions, and suitable tensile strength. The mesh must allow tissue to grow, peritoneal regeneration, and the regular healing process [7].

The high prevalence of parastomal hernia and unsatisfactory clinical results make this ailment challenging to treat. The prophylactic use of a prosthetic mesh is one of the most increasingly used approaches and may reduce the frequency of the occurrence of parastomal hernia [8].

The aim of this work is to outline the problems related to parastomal hernia, review the key properties of surgical meshes available on the market, present surgical techniques currently used to treat parastomal hernia, and characterize the related complications.

## 2. Parastomal Hernia

Stoma is a surgically created connection between an organ’s lumen and the body’s surface, i.e., the skin. Stomas are defined depending on the organ involved: colostomy—colon fistula; ileostomy—small intestine fistula; urostomy—urinary tract fistula [9].

The most common long-term complication of a stoma is a parastomal hernia, occurring in approximately 50% of patients with an isolated stoma [10,11]. It is estimated that this is an inevitable complication, and the risk of hernia increases by several percent every year after stoma surgery [12]. According to Carne et al., the incidence of parastomal hernia is assessed differently depending on the type of stoma: with loop ileostomy, hernia occurs in about 0–6.2% of cases; with loop colostomy, hernia occurs in about 0–30.8% of cases; with ileostomy, hernia occurs in approximately 1.8–28.3% of cases, and with colostomy, hernia occurs in 4–48.1% of cases [13].

There are several classifications of parastomal hernia, but so far, the most widely applied is the classification created by a team led by Professor Marek Szczepkowski MD, PhD, named the Bielanski Hospital Classification (BHC) (Table 1) [14]. Based on this classification, the European Hernia Society (EHS) classification was subsequently developed.

According to the clinical indications and types of hernia, a parastomal hernia should be treated as a special case of incisional hernia. The development of a hernia is the result of alternations within the structure of the connective tissue, including a problem with collagen synthesis [15,16,17].

## 3. Treatment

In Poland, according to National Health Fund (NFZ) data, about 40,000 patients live with a stoma, and each year, approximately 7000 stoma operations are performed, at least 2000 of which will be a parastomal hernia that is eligible for surgical repair [18].

Polish summaries published by the National Health Fund within the “Statistics of the NFZ JGP” programs were analyzed. The listed stoma procedures in which a mesh implant can be applied are presented in Table 2.

The above treatments from groups F22, F31 (A), and F32 account for 10.3% of all procedures, leading to, on average, 4323 hospitalizations per year. The mean value of hospitalization in these groups is EUR 2436. The estimated total value of hospitalizations in these groups is approximately EUR 10.5 million [19].

Repair with the use of a mesh implant (mesh graft) is currently the standard procedure used in the treatment of parastomal hernias. The previously recommended simple suturing of the fascial defect and translocation of the stoma have now been almost completely withdrawn due to the high percentage of hernial recurrences (33–76%) [20] and local infection at a level of 12% [21,22,23].

The method for treating a hernia depends on the extent of damage of the fascia muscular elements of the abdominal wall, the presence of an infection focus, the possibility of using appropriate prosthetic materials, the patient’s condition, and the preferences and qualifications of the doctor [24].

The use of a synthetic mesh significantly reduces the recurrence rate of parastomal hernias, but the rate of local complications still requires attention and ranges from 7% to 18% [23].

### 3.1. Surgical Access

Open and laparoscopic surgery is possible for all types of hernia. The decision regarding the type of surgery is made based on the surgeon’s preferences and the patient’s medical history and comfort. Laparoscopic surgery is associated with less pain, shorter hospitalization time, and a low probability of intraoperative complications; on the other hand, it requires general anesthesia. People with massive intraperitoneal adhesions, past inflammatory processes of the abdominal cavity, or radiotherapy will not benefit from the laparoscopic technique. For open surgery, the procedure is easier to perform and can be performed under local anesthesia but is characterized by a longer hospitalization time and a greater number of complications [25].

A study by Halabi and colleagues showed that only 10.4% of patients in the study underwent laparoscopic parastomal hernia surgery. The authors concluded that this result may be related to the high number of adhesions in the case of stoma hernias or clear clinical evidence for the management of this type of hernia [26].

### 3.2. Implant Position

The promising results in using mesh implants for other hernia types [27,28,29,30] have encouraged the implantation of meshes also in the treatment of parastomal hernia and for prophylactic purposes.

Studies conducted on various methods of mesh arrangements—sublay, onlay, and intraperitoneal onlay mesh (IPOM)—during parastomal hernia surgery confirmed the minimization of intraoperative and postoperative complications after implantation of a mesh device in comparison with conventional suture repair [31,32].

The simplest method for the use of a synthetic implant is to place the prosthesis on the fascia in the “onlay” position. This procedure involves first preparing the hernial sac and its contents and then reducing or removing the sac after opening. Then, after suturing the hernial gates, an appropriately selected mesh size is placed on the fascia [24]. The theoretical advantage of this technique is that patients do not need to undergo extensive dissection of the abdominal wall to create planes in which the mesh can be inserted, resulting in a shorter recovery time. The disadvantage is that the pressure in the abdominal cavity can displace the mesh, which increases the risk of recurrence, reported in up to 18.6% of cases [23]. Another disadvantage of the onlay method is the increased risk of infection because the selected implant is located near the contaminated stomal opening.

An alternative method is the “sublay” technique, which involves placing the mesh in the preperitoneal layer and fixing it with a single non-absorbable suture. The sublay method is characterized by a lower risk of infection and greater stabilization of the mesh via intra-abdominal pressure [24]. While sublay repair prevents the mesh from interacting with the abdominal organs, the intraperitoneal position (IPOM) poses an increased risk of intestinal erosion and the formation of adhesions. During IPOM repair, care should be taken to maximize tissue adhesion between the mesh and the abdominal wall to minimize seroma formation.

Hansson et al. systematically reviewed surgical techniques for parastomal hernia repair, including a total of 35 studies, which found onlay to have the highest recurrence rate and IPOM to have the lowest [23].

### 3.3. Operational Technique

In the case of parastomal hernia, the applied surgical technique has a key influence on the number of recurrences [23].

Among the techniques for the repair of parastomal hernia with the use of a mesh, the most common in the literature are the Sugarbaker technique (or the modified Sugarbaker technique using a laparoscopic approach), the keyhole technique, and the sandwich technique.

In the modified Sugarbaker technique, intraperitoneal adhesions and hernial gates are prepared after generation of the pneumoperitoneum. After dissecting the adhesions, the intestine is moved to the lateral edge of the hernial gate to create a tunnel between the parietal peritoneum and the mesh. After inserting the mesh into the peritoneal cavity, the hernial gates are covered with a margin of at least 6–7 cm in all directions or, if necessary, across all coexisting hernias. The mesh is fixed to the parietal peritoneum using tackers or sutures at intervals of 3–4 cm [33].

Open access and laparoscopic access are also used for so-called keyhole surgery. This technique uses a round or oval mesh cut from the medial side with an opening for the stoma. This mesh is inserted intraperitoneally. After being fixed, the mesh is sutured, which leads to its closure around the stoma [34,35].

The third technique is the “sandwich” technique, which is a combination of the Sugarbaker and “keyhole” techniques and uses two meshes that are placed intraperitoneally. The first implant, which is the incised mesh or the hole-type mesh, is placed around the stoma sling to cover the orifice of the hernia using the “keyhole technique”. The second flat mesh implanted by the Sugarbaker technique forms a plane and, with its medial edge, covers the median wound after laparotomy, making it possible to supply the hernia through the postoperative scar in parallel.

The relapse rate for the Sugarbaker technique is 11.6%, that of the keyhole technique is 34.6% [34], and that of the sandwich technique is 2.1% [36].

The latest research describes experiments that use a dedicated synthetic composite mesh with a 3D structure (Dynamesh—IPST) for the prevention of parastomal hernia. This observational study included 88 patients divided into two cohort groups of patients: a mesh prophylaxis group (43 patients) and a non-mesh prophylaxis group (45 patients). The implant was placed in the IPOM position. During the procedure, the intestinal loop was pulled through a central funnel with 2–3 cm diameter. Then, the funnel was oriented towards the abdomen and made to fit snugly around the gut to prevent stomal loss as well as hernia recurrence. The mesh was fixed with tackers, anchors, or surgical sutures. The results of the study showed that parastomal hernia occurrence after 12 months was 11% in the mesh prophylaxis group (MP) and 54% in the non-mesh prophylaxis group (NMP). There were no significant differences in long-term complications (bowel occlusion (2—MP; 0—NMP), stenosis (2—MP; 1—NMP), or prolapse (1—MP; 2—NMP)) [37]. 

The aim of the study by G. Kohler et al. was to assess the occurrence of postoperative complications and the possibility of parastomal hernia in patients undergoing stoma surgery with simultaneous prophylactic placement of a 3D hernia mesh. The retrospective analysis was based on the collected data of 80 patients. A parastomal hernia developed in three patients (3.75%). No mesh-related complications were reported. In seven patients, there were complications related to the emerged stoma (infections (3), seroma (2), stenosis (1), and stomal retraction (1)). According to the authors, using the prophylactic implantation of a specially selected 3D mesh implant via the IPOM technique, stoma formation is safe, efficient, and relatively easy to perform. In contrast to flat meshes, by using meshes featuring chimneys at the boundary areas of the stoma, the stoma can be well protected against the internal organs. At the same time, the vertical funnel-shaped portion of the mesh provides protection against stoma loss [38].

Another publication compared the methods for treating a parastomal hernia, focusing on the complications that arose and the number of relapses (Table 3). The analysis performed included 135 patients, and eight different surgical techniques were used. Laparoscopic operations accounted for 46.7% (63/135). In 44 cases, the hernia recurred (32.6%), while in 24 patients (17.8%), perioperative complications occurred; 12 of these patients underwent re-operation. Only in the case of hernia repair using an implant with a 3D structure featuring both open and laparoscopic access were no hernia recurrences noted [39].

A review carried out by Francis J DeAsis et al. focused on evaluating the efficacy and safety of laparoscopic approaches for parastomal hernia repair. A total of 469 patients were deemed eligible for the present review. Three different surgical techniques were described. Most studies used an expanded polytetrafluoroethylene (ePTFE) mesh. The primary outcome analyzed was the recurrence of parastomal hernia. The recurrence rate was 10.2% for the modified laparoscopic Sugarbaker approach, whereas the recurrence rate was 27.9% for the keyhole approach. For the sandwich technique, there was one recurrence out of 47 repairs. Secondary outcomes (referring to the overall cohort) were mesh infection (1.7%), surgical site infection (3.8%), obstruction requiring reoperation (1.7%), and other complications, such as ileus, pneumonia, and urinary tract infections (16.6%). There were no intraoperative mortalities and six mortalities during the postoperative course [40].

## 4. Mesh Implants 

In addition to the variety of surgical techniques used in the treatment of parastomal hernias, a number of materials are currently available for the replacement of fascia muscular defects in the abdominal wall [24].

Regardless of the type of material used, the material should meet a number of basic parameters that affect the body’s immune response to the implant and also reduce fibrosis [41,42].

In the repair of abdominal hernias, prostheses are characterized by their durability and ease of use. The construction of mesh devices used in the surgical treatment of parastomal hernia should allow for safe intraperitoneal implantation via open and laparoscopic methods [24].

### 4.1. Required Parameters and Properties

#### 4.1.1. Resistance and Elasticity

The tension on the abdominal wall can be calculated according to Laplace’s law: tension = (diameter × pressure)/(4 × wall thickness). The maximum intra-abdominal pressure in healthy adults is generated by coughing and jumping and is approximately 170 mmHg. Surgical meshes used to repair large hernias should withstand at least 180 mmHg before they break (tensile strength approx. 16 N/cm) [41,42].

Notably, the abdominal wall has twice the flexibility in the longitudinal direction than in the transverse direction [43,44].

The overall strength of the implant depends on the material used. Hernia recurrence increases when the mesh stretches more or less than the abdominal wall. Moreover, to meet the elasticity requirements, a hernial mesh should be elastic in all directions and have an anisotropic structure [45]. 

Saberski and Novitsky evaluated the anisotropy of six commercial meshes: Prolite, Pareietex, Ultrapro, Trelex (made of polypropylene), Dualmesh ePTFE, and INFINIT knitted polytetrafluoroethylene (PTFE). All implants, except for Dualmesh, feature significant anisotropic properties [46].

Due to the anisotropic properties of most of the designed products, there is a need to describe the two-direction strength properties of the meshes on the packaging, as well as the lowest strength value.

#### 4.1.2. Pore Size

Porosity is a major indicator of tissue response. The geometry and sizes of pores define the ability of mesh ingrowth. These parameters can be controlled during the manufacturing process. Mesh pores are classified into five different groups: micro-pores (size less than 0.1 mm), small pores (0.1–0.6 mm), medium pores (1 mm), large pores (1–2 mm), and very large pores (>2 mm) [47].

The pores of meshes must be larger than 75 µm to allow the infiltration/penetration of macrophages, fibroblasts, blood vessels, and collagen. Surgical meshes with larger pores allow for the faster growth of soft tissues and are also more flexible due to the lack of formation of so-called granuloma bridging [30,41,48].

The above-mentioned bridges illustrate the process by which individual granulomas blend together and cover the entire mesh. As a result, the mesh stiffens the implant and reduces its flexibility. This phenomenon occurs in meshes with pores smaller than 800 µm [41].

Studies have shown that macroporous meshes have a positive effect on vascularization [30] and also reduce the risk of infection [49]. Meshes with larger pore sizes featuring reduced polypropylene mass lead to a decreased inflammatory reaction in the human abdominal wall [50]. 

Orenstein et al. reported that large pore sizes (pores larger than 1.5 mm) in meshes improved patients’ quality of life after a surgical procedure with the mesh [51].

According to the review carried out by A.S.W. Jacombs, the dual concepts of effective porosity and biofilm may be crucial in mesh-related morbidity and should be investigated further. Developing new mesh implants to maintain effective porosity and reduce biofilm formation may help reduce mesh-related complications [52].

#### 4.1.3. Surface Mass

The weight of the surgical mesh depends both on the weight of the biomaterial itself and the amount of its use (pore size). Heavy surgical meshes are made out of materials with a higher linear mass and have both small pores and high tearing strength. On the other hand, ultralight surgical meshes are made from thinner filaments and have large pores (>1 mm). Lightweight meshes, in general, reduce adverse effects, including chronic pain, fibrosis, adhesion and inflammatory response, and foreign body sensation. These meshes are also characterized by greater flexibility in comparison with heavy-weight meshes [53,54,55,56]. Despite their lower strength parameters, ultralight meshes are able to withstand pressures above the maximum intra-abdominal pressure of 170 mmHg.

Based on their weights, the meshes are classified into four categories: ultralight meshes (<35 g/m^2^), light meshes (≥35–70 g/m^2^), standard meshes (≥70–140 g/m^2^), and heavy meshes (>140 g/m^2^) [44,57,58].

#### 4.1.4. Implant Contraction

Shrinkage of the mesh occurs due to the reduction in scar tissue around it. Scar tissue contracts to approximately 60% of the original wound surface, and smaller pores cause greater contraction [41].

It is assumed that light meshes, which reduce the amount of scar tissue formation, will have a lower degree of contraction [48]. Due to this shrinkage, it is recommended to use at least a 5-cm overlap around the defect [59].

Research carried out by Masayuki Endo et al. showed a reduction in the apparent surface area between implantation and the second day, indicating that most mesh deformation occurs prior to tissue ingrowth. In conclusion, the surface area of implanted meshes (Marlex, DynaMesh, Ultrapro) decreased by 21% by day 90 [60].

#### 4.1.5. Biocompatibility

The biomaterials currently available on the market are physically and chemically inert. They are generally stable, non-toxic, and do not trigger an immune system response. Despite this, these materials are not biologically inert, and their presence in the patient’s body causes a reaction to the foreign body (inflammation, fibrosis, calcification, thrombosis, and the formation of granulomas). Mesh implantation infections are always of concern because they are difficult to deal with without removing the mesh. The risk of infection increases in an infected surgical field, such as parastomal hernia surgery.

#### 4.1.6. Adhesion to the Intestines

As the medical community began to locate the surgical mesh, concerns arose about the adhesion of this device. The effect on the adhesion of the implant to the intestine is determined by the structure and surface of the fibers and the size of the pores. Meshes with a high surface mass cause intense fibrotic processes, yielding strong adhesion to the abdominal wall [61].

On the other hand, microporous ePTFE meshes do not allow for tissue hypertrophy. Therefore, the risk of adhesion is lower, as the mesh is unable to adhere to the abdominal wall [62].

The presented examples show the difficulties associated with the production of a surgical mesh that would have both adhesive and anti-adhesive properties. Hence, manufacturers of surgical meshes have made attempts to design a composite device that allows the mesh to grow into the abdominal cavity without the formation of adhesions on the peritoneal side through the use of surface modification techniques and methods of knitting fabrics by combining several synthetic materials.

#### 4.1.7. Material

For the production of surgical meshes, monofilament or multifilament yarns of various surface masses are used [63,64]. Microporous and multifilament meshes are among the devices at a higher risk of infection because macrophages and neutrophils are unable to penetrate through small pores (<10 µm). This allows bacteria (<1 µm) to survive unhindered inside the pores. Meshes with a low infection risk include those made of monofilaments with openings greater than 75 µm [65].

### 4.2. Implants Available on the Market

#### 4.2.1. Synthetic

Non-resorbable synthetic surgical meshes are used for long-term strengthening of damaged tissues as a result of a hernia. Mesh implants come in two different structures: knitted and woven. The conducted research showed that polypropylene (PP) is the most commonly used non-absorbable material for mesh implants [41,66,67,68].

Apart from PP, the materials used also include ePTFE, PTFE, and poly(ethylene terephthalate) (PET) [69,70].

First-generation implants can be divided into three categories: (1) macroporous meshes, (2) microporous meshes, (3) and macroporous meshes with multifilament and microporous components [71].

The use of non-resorbable mesh implants has many clinically proven advantages and disadvantages. On the one hand, due to the higher strength values of these meshes compared to natural tissues, these meshes lead to a reduction in flexibility, the occurrence of immune reactions in the body, and chronic pain. On the other hand, their affordable price and good overgrowth with native tissue support their worldwide use [55].

First-generation implants also include fully resorbable meshes. Unlike non-resorbable meshes, these meshes are designed to minimize inflammation and the amount of foreign material implanted. Due to progressive degradation, mechanical stability can be lost early, leading to possible hernia recurrences [65]. Table 4 presents examples of first-generation mesh implants available on the market. 

#### 4.2.2. Composite

Second-generation meshes include implants made of at least two synthetic materials with different properties on each side of the mesh (Table 5). Improvements have been made to reduce complications such as recurrent hernia, infections, and adhesions. The main advantage of composite meshes is their intraperitoneal application. The side of the mesh in contact with the peritoneum is usually smooth and microporous to prevent adherence to the intestines, while the side facing the connective tissues is rough with large pores, which positively affects tissue overgrowth [72].

Composite meshes mainly consist of a PP core covered with another synthetic or natural material. Materials used for non-adhesive coatings include, among others, titanium [73,74], chitosan [75], poly(glycolic acid) (PGA) [76], cellulose [77], and collagen [78]. Another approach for the production of composite meshes is to join filaments with different properties, such as PP, e-PTFE [79], and polyvinylidene fluoride (PVDF) [80,81].

Experience in the use of composite meshes for both repair and prophylactic purposes in parastomal hernia indicates a minimal risk of infection and a low risk of complications [82].

#### 4.2.3. Biological

Biological meshes were introduced in the 1990s. Cells derived from human tissues (allograft) or animals (xenograft) are used for their production. The dermis is the most commonly used tissue due to its ability to create a larger mesh size, but prostheses with cells derived from the intestinal mucosa and pericardium are also available. Essentially, all biological meshes provide the extracellular scaffold necessary to rebuild healthy tissue, allowing mass transport by ingrowing new blood vessels and infiltrating native cells, including fibroblasts and myocytes, ultimately resulting in the deposition of a new extracellular matrix [83].

Compared to synthetic meshes, biological meshes are more biocompatible and elicit a lower inflammatory response in the body but are associated with a greater number of hernia recurrences due to their lower mechanical strength compared to synthetic meshes. In a clinical trial comparing PP and biological meshes, 12% of patients experienced a recurrence of hernia after implantation of a biological mesh, but no recurrence was observed with the synthetic mesh [84]. Commercially available biological mesh implants are outlined in Table 6. 

## 5. Prophylactic Implantation of a Mesh Device

According to the guidelines of the European Hernia Society, the prevention of parastomal hernias in patients undergoing end colostomy surgery with prophylactic mesh implantation was satisfactory [89].

The prophylactic use of a mesh implant in permanent stoma surgery reduces the risk of a parastomal hernia by 75%. Moreover, complications occur only in individual cases, so it can be concluded that mesh implantation in this type of surgery could be routinely applied [90].

An analysis conducted by Shuanhu Wang et al. aimed at assessing the effectiveness of prophylactic mesh implantation during end colostomy. The results showed that in the case of sigmoid terminal colostomy, prophylactic mesh placement reduced the incidence of parastomal hernias and associated reoperations. There were no significant differences in stoma-related complications. Moreover, the surgical techniques of sublay and IPOM are considered to be safe and feasible, reducing the likelihood of a parastomal hernia [91].

## 6. Current Trends

Electrospinning and 3D printing are examples of manufacturing techniques used for the fabrication of drug-loaded devices.

The encapsulation of antimicrobial agents or drugs is one of the possible approaches that could be utilized to produce meshes with antibacterial properties. Pérez-Köhler et al. developed a new coating material known as hyaluronic acid-poly(N-isopropylacrylamide) (HApN), which forms a hydrogel that can be used as a coating for meshes only when it reaches body temperature. The authors selected two different coating formulations—one based on antibiotics (gentamicin + rifampicin) and one based on an antiseptic (chlorhexidine). The results of this study showed that HApN, when loaded with drugs, inhibited the in vitro the growth of several Gram-positive and Gram-negative bacteria [92]. 

The next study carried out by Nadia Qamar et al. explored the application of the fused deposition modeling in the fabrication of personalized hernial meshes with and without loading of a pharmaceutical agent (ciprofloxacin HCl). All the printed meshes (PP and polyvinyl alcohol (PVA)) showed good mechanical properties. Meshes made of PVA demonstrated a faster release of the loaded drug in comparison to the PP mesh. Moreover, in vivo testing revealed no signs of implant rejection along with a reduction in adhesion to the visceral side and faster wound healing [93].

Another solution to improve the implant properties could involve the use of metallic or diamond nanoparticles. A polypropylene–nano-diamond composite hernia mesh exhibited a significant reduction in protein absorption consistent with lower inflammatory responses; furthermore, no cytotoxicity was observed [94].

The implementation of these novel materials needs further clinical trials to determine the superiority of such materials compared to those available on the market.

## 7. Conclusions

The best strategy for the prevention and treatment of parastomal hernias has not yet been identified. The variety of available mesh implants—their various sizes, materials, possible spatial structures, and related surgical techniques—and the choices of mesh arrangement in relation to the layers of the abdominal wall allow surgeons to choose the best parameters depending on the preferences and needs of the patient. Choosing the right surgical mesh, however, is not the objective for a successful operation. One of the most important considerations for surgeons should be the technique used to secure the mesh in the surgical field. If the mesh is too small or under too much tension, complications with implantation will be inevitable, regardless of the material used. Despite the reduction in hernia recurrence when surgical meshes are used, it remains necessary to consider the possibility of complications such as infections, adhesions, or intestinal obstruction. Most of these disadvantages are related to the chemical and structural nature of the implant itself. The “golden mean” for the mesh is considered to be optimal integration with the abdominal wall and minimal adherence on the peritoneal side. Due to the technique’s satisfactory clinical results, the prophylactic use of meshes during stoma recovery is an increasingly common approach to reduce the occurrence of parastomal hernias.

Despite the progress that has been made in the design of hernial meshes, further research is needed to understand the complex tissue–implant interactions to achieve a reduction in adhesion, infections, and immune responses, as well as better biocompatibility.

## Figures and Tables

**Table 1:** Bielanski Hospital Classification (BHC) Classification.

	BHC Classification
Type I	Small, isolated parastomal hernia
Type II	Parastomal hernia with coexisting hernia in the midline scar (no significant abdominal deformation)
Type III	Isolated large parastomal hernia
Type IV	Parastomal hernia with coexisting hernia in a scar after a midline incision (significant abdominal deformity)

**Table 2:** Stoma procedures in which a mesh implant can be applied.

Surgical Procedure	Group
Loop ileostomy	F22
Other ileostomy	F22
Permanent ileostomy—other	F22
Anterior rectal resection with the formation of a colostomy	F31 (A)
Permanent colostomy	F32
Colostomy—other	F32
Loop colostomy	F32

**Table 3:** List of complications depending on the method used [39].

Surgical Technique	Complication Occurrence (Re-Operation)	Hernia Recurrence (Re-Operation)	Number of Performed Treatments
Tension method	4 (3)	10 (6)	25
Onlay	5 (4)	12 (6)	22
Sublay	3 (1)	9 (8)	20
Laparoscopic method: keyhole	4 (2)	10 (6)	22
Laparoscopic method: Sugarbaker	1 (1)	2 (2)	4
Laparoscopic method: “sandwich”	4 (1)	1 (0)	21
Laparoscopic method with 3D implant	2 (0)	0	16
Laparotomy method with 3D implant	1 (0)	0	5

**Table 4:** Classification of commercially available first-generation mesh implants [41].

Product	Manufacturer	Material	Filament	Surface Mass (g/m^2^)	Resorbable	Pore Size (mm)
Vicryl	Ethicon	Polyglactin	Multifilament	56	Yes	0.4
Sefil	B-Baun	PGA	Multifilament	56	Yes	0.75
Goretex	Gore	e-PTFE	Multifilament	N.A.	No	0.003
Optomesh	Tricomed	PP	Monofilament	60–85	No	>1
Parietene	Covidien	PP	Monofilament	80–100	No	0.8
Prolene	Ethicon	PP	Monofilament	80–100	No	0.8
3D Max	BARD	PP	Monofilament	80–100	No	0.8
Premilene	B-Braun	PP	Monofilament	80–100	No	0.8
Polysoft	BARD	PP	Multifilament	80–100	No	0.8
Optomesh Ultralight	Tricomed	PP	Monofilament	24–35	No	>1
Prolene Light	Covidien	PP	Monofilament	36–48	No	1.0–3.6
Optilene	B-Baun	PP	Monofilament	36–48	No	1.0–3.6
Mersilene	Ethicon	PP	Monofilament	40	No	1.0–2.0
Parietex Lightweight	Medtronic	PET	Monofilament	N.A.	No	>1

**Table 5:** Classification of commercially available composite mesh implants [41].

Product	Manufacturer	Material	Filament	Surface Mass (g/m^2^)	Resorbable	Pore Size
Ultrapro	Ethicon	PP/PGC-25	Monofilament	28	Partially (<140 days)	>3
Vypro, Vypro II	Ethicon	PP/polyglactin 910	Multifilament	25 and 30	Partially (42 days)	>3
Composix EX Dulex	BARD	PP/e-PTFE	Monofilament	N.A.	No	0.8
Proceed	Ethicon	PP/cellulose	Monofilament	45	Partially	N.A
TiMeshTiMesh Extralight	PFM	PP/tytan	Monofilament	16 and 35	No	>1
DynaMesh—IPST/IPOM	FEG Textiltechnik	PP/PVDF	Monofilament	60	No	1–2

**Table 6:** Classification of commercially available biological mesh implants [85,86,87,88].

Product	Manufacturer	Material	Cross-Linking	Resistance (MPa)
CollaMend	Davol	Animal cell-free skin matrix	Yes	11
Permacol	Covidien	Animal cell-free skin matrix	Yes	39
Strattice	LifeCell	Animal cell-free skin matrix	No	18
XenMatrix	Davol	Animal cell-free skin matrix	No	14

## Data Availability

The data presented in this study are openly available at [doi:10.1007/s00268-015-3187-1], [39]; at [doi:10.1308/003588410 × 12664192076296], [41]; at [doi.org/10.1007/s10029-013-1054-2], [85]; at [doi:10.1007/s10029-013-1070-2], [86]; at [doi:10.1007/s10029-010-0777-6], [87]; at [doi:10.1016/j.surge.2012.02.006], [88].
