# Correction: Turlakiewicz et al. The Role of Mesh Implants in Surgical Treatment of Parastomal Hernia. *Materials* 2021, *14*, 1062

**DOI:** 10.3390/ma14133511

**Published:** 2021-06-24

**Authors:** Karolina Turlakiewicz, Michał Puchalski, Izabella Krucińska, Witold Sujka

**Affiliations:** 1Institute of Material Science of Textiles and Polymer Composites, Lodz University of Technology, Żeromskiego 116, 90-924 Lodz, Poland; michal.puchalski@p.lodz.pl (M.P.); izabella.krucinska@p.lodz.pl (I.K.); 2Tricomed S.A., Świętojańska 5/9, 93-493 Lodz, Poland; witold.sujka@tzmo-global.com

We have recently been made aware by Dr. Holmdahl’s (Umeå University—Department of Surgical and Perioperative Sciences) and the MDPI Editorial offices of some errors and omissions in Section 4.2.3. Biological of our recent paper [1]. The second paragraph of said Section 4.2.3. Biological currently reads as follows:

(Compared to synthetic meshes, biological meshes are more biocompatible and elicit a lower inflammatory response in the body but are associated with a greater number of hernia recurrences due to their lower mechanical strength compared to synthetic meshes. In a clinical trial comparing PP and biological meshes, 12% of patients experienced a recurrence of hernia after implantation of a biological mesh, but no recurrence was observed with the synthetic mesh [84]. Commercially available biological mesh implants are outlined in Table 6.)

To set straight the scientific record, we would like to make the following corrections:

(Compared to synthetic meshes, biological meshes are more biocompatible and elicit a lower inflammatory response in the body but are associated with a greater number of hernia recurrences due to their lower mechanical strength compared to synthetic meshes [84]. Although, the research carried out by Holmdahl et. al. showed comparable recurrence rates between the group of patients who received a full-thickness skin graft (8.3%) and a synthetic mesh (7.1%) [85]. Commercially available biological mesh implants are outlined in Table 6.)

Adding a new article resulted in the renumbering of the bibliography in the manuscript. Below amendments were made:

**Table 6 materials-14-03511-t006:** Classification of commercially available biological mesh implants [86–89].

Product	Manufacturer	Material	Cross-Linking	Resistance (MPa)
CollaMend	Davol	Animal cell-free skin matrix	Yes	11
Permacol	Covidien	Animal cell-free skin matrix	Yes	39
Strattice	LifeCell	Animal cell-free skin matrix	No	18
XenMatrix	Davol	Animal cell-free skin matrix	No	14

## 5. Prophylactic Implantation of a Mesh Device

According to the guidelines of the European Hernia Society, the prevention of parastomal hernias in patients undergoing end colostomy surgery with prophylactic mesh implantation was satisfactory [90].

The prophylactic use of a mesh implant in permanent stoma surgery reduces the risk of a parastomal hernia by 75%. Moreover, complications occur only in individual cases, so it can be concluded that mesh implantation in this type of surgery could be routinely applied [91].

An analysis conducted by Shuanhu Wang et al. aimed at assessing the effectiveness of prophylactic mesh implantation during end colostomy. The results showed that in the case of sigmoid terminal colostomy, prophylactic mesh placement reduced the incidence of parastomal hernias and associated reoperations. There were no significant differences in stoma-related complications. Moreover, the surgical techniques of sublay and IPOM are considered to be safe and feasible, reducing the likelihood of a parastomal hernia [92].

## 6. Current Trends

Electrospinning and 3D printing are examples of manufacturing techniques used for the fabrication of drug-loaded devices.

The encapsulation of antimicrobial agents or drugs is one of the possible approaches that could be utilized to produce meshes with antibacterial properties. Pérez-Köhler et al. developed a new coating material known as hyaluronic acid-poly(N-isopropylacrylamide) (HApN), which forms a hydrogel that can be used as a coating for meshes only when it reaches body temperature. The authors selected two different coating formulations—one based on antibiotics (gentamicin + rifampicin) and one based on an antiseptic (chlorhexidine). The results of this study showed that HApN, when loaded with drugs, inhibited the in vitro the growth of several Gram-positive and Gram-negative bacteria [93]. 

The next study carried out by Nadia Qamar et al. explored the application of the fused deposition modeling in the fabrication of personalized hernial meshes with and without loading of a pharmaceutical agent (ciprofloxacin HCl). All the printed meshes (PP and polyvinyl alcohol (PVA)) showed good mechanical properties. Meshes made of PVA demonstrated a faster release of the loaded drug in comparison to the PP mesh. Moreover, in vivo testing revealed no signs of implant rejection along with a reduction in adhesion to the visceral side and faster wound healing [94].

Another solution to improve the implant properties could involve the use of metallic or diamond nanoparticles. A polypropylene–nano-diamond composite hernia mesh exhibited a significant reduction in protein absorption consistent with lower inflammatory responses; furthermore, no cytotoxicity was observed [95].

The implementation of these novel materials needs further clinical trials to determine the superiority of such materials compared to those available on the market.

**Data Availability Statement:** The data presented in this study are openly available at [doi:10.1007/s00268-015-3187-1], [39]; at [doi:10.1308/003588410X12664192076296], [41]; at [doi.org/10.1007/s10029-013-1054-2], [86]; at [doi:10.1007/s10029-013-1070-2], [87]; at [doi:10.1007/s10029-010-0777-6], [88]; at [doi:10.1016/j.surge.2012.02.006], [89].

## References

84.Wang See, C.; Kim, T.; Zhu, D. Hernia Mesh and Hernia Repair: A Review. *Eng. Regen.*
**2020**, *1*, 19–33.85.Holmdahl, V.; Stark, B.; Clay, L.; Gunnarsson, U.; Strigard, K. One-year out-come after repair of giant incisional hernia using synthetic mesh or full- thickness skin graft: A randomised controlled trial. *Hernia* **2019**, *23*, 355–361.86.Alicuben, E.T.; Demeester, S.R. Onlay ventral hernia repairs using porcine non-cross-linked dermal biologic mesh. *Hernia* **2013**, *18*, 705–712.87.Cavallo, J.A.; Greco, S.C.; Liu, J.; Frisella, M.M.; Deeken, C.R.; Matthews, B.D. Remodeling characteristics and biomechanical properties of a crosslinked versus a non-crosslinked porcine dermis scaffolds in a porcine model of ventral hernia repair. *Hernia* **2013**, *19*, 207–218.88.Mulier, K.E.; Nguyen, A.H.; Delaney, J.P.; Marquez, S. Comparison of Permacol™ and Strattice™ for the repair of abdominal wall defects. *Hernia* **2011**, *15*, 315–319.89.Smart, N.J.; Marshall, M.; Daniels, I.R. Biological meshes: A review of their use in abdominal wall hernia repairs. *Surgery* **2012**, *10*, 159–171.90.Antoniou, S.A.; Agresta, F.; Alamino, J.M.G.; Berger, D.; Berrevoet, F.; Brandsma, H.; Bury, K.; Conze, J.; Cuccu-rullo, D.; Dietz, U.A.; et al. European Hernia Society guidelines on prevention and treatment of parastomal hernias. *Hernia* **2018**, *22*, 183–198.91.Cross, A.J.; Buchwald, P.L.; Frizelle, F.A.; Eglinton, T.W. Meta-analysis of prophylactic mesh to prevent parastomal hernia. *BJS*
**2017**, *104*, 179–186.92.Wang, S.; Wang, W.; Zhu, B.; Song, G.; Jiang, C. Efficacy of Prophylactic Mesh in End-Colostomy Construction: A Systematic Review and Meta-analysis of Randomized Controlled Trials. *World J. Surg.* **2016**, *40*, 2528–2536.93.Pérez-Köhler, B.; Linardi, F.; Pascual, G.; Bellón, J.M.; Eglin, D.; Guillaume, O. Efficacy of antimicrobial agents delivered to hernia meshes using an adaptable thermo-responsive hyaluronic acid-based coating. *Hernia* **2020**, *24*, 1201–1210.94.Qamar, N.; Abbas, N.; Irfan, M.; Hussain, A.; Arshad, M.S.; Latif, S.; Mehmood, F.; Ghori, M.U. Personalized 3D printed ciprofloxacin impregnated meshes for the management of hernia. *J. Drug Deliv. Sci. Technol.* **2019**, *53*, 101164.95.Houshyar, S.; Sarker, A.; Jadhav, A.; Kumar, G.S.; Bhattacharyya, A.; Nayak, R.; Shanks, R.A.; Saha, T.; Rifai, A.; Padhye, R.; et al. Polypropylene-nanodiamond composite for hernia mesh. *Mater. Sci. Eng. C*
**2020**, *111*, 110780.

These changes have no material impact on the conclusions of our paper. We apologize for any inconvenience caused to the readers.

## References

[1] Turlakiewicz K., Puchalski M., Krucińska I., Sujka W. (2021). The Role of Mesh Implants in Surgical Treatment of Parastomal Hernia. Materials.

